# Evaluation of GFP reporter utility for analysis of transcriptional slippage during gene expression

**DOI:** 10.1186/s12934-018-0999-3

**Published:** 2018-09-21

**Authors:** Ewa Wons, Dawid Koscielniak, Monika Szadkowska, Marian Sektas

**Affiliations:** Department of Microbiology, University of Gdansk, Wita Stwosza 59, 80-308 Gdansk, Poland

**Keywords:** Green fluorescent protein reporter, Transcriptional slippage, A/T homopolymers, T7 RNA polymerase, *E. coli* RNA polymerase

## Abstract

**Background:**

Epimutations arising from transcriptional slippage seem to have more important role in regulating gene expression than earlier though. Since the level and the fidelity of transcription primarily determine the overall efficiency of gene expression, all factors contributing to their decrease should be identified and optimized.

**Results:**

To examine the influence of A/T homopolymeric sequences on introduction of erroneous nucleotides by slippage mechanism green fluorescence protein (GFP) reporter was chosen. The in- or out-of-frame *gfp* gene was fused to upstream fragment with variable number of adenine or thymine stretches resulting in several hybrid GFP proteins with diverse amino acids at N-terminus. Here, by using T7 phage expression system we showed that the intensity of GFP fluorescence mainly depends on the number of the retained natural amino acids. While the lack of serine (S_2_) residue results in negligible effects, the lack of serine and lysine (S_2_K_3_) contributed to a significant reduction in fluorescence by 2.7-fold for polyA-based in-frame controls and twofold for polyTs. What is more, N-terminal tails amino acid composition was rather of secondary importance, since the whole-cell fluorescence differed in a range of 9–18% between corresponding polyA- and polyT-based constructs.

**Conclusions:**

Here we present experimental evidence for utility of GFP reporter for accurate estimation of A/T homopolymeric sequence contribution in transcriptional slippage induction. We showed that the intensity of GFP hybrid fluorescence mainly depends on the number of retained natural amino acids, thus fluorescence raw data need to be referred to appropriate positive control. Moreover, only in case of GFP hybrids with relatively short N-terminal tags the fluorescence level solely reflects production yield, what further indicates the impact of an individual slippage sequence. Our results demonstrate that in contrast to the *E. coli* enzyme, T7 RNA polymerase exhibits extremely high propensity to slippage even on runs as short as 3 adenine or 4 thymine residues.

**Electronic supplementary material:**

The online version of this article (10.1186/s12934-018-0999-3) contains supplementary material, which is available to authorized users.

## Background

Green fluorescent protein (GFP) from the *Aequorea victoria* jellyfish [1] is nowadays the most widely used and developed reporter applicable in biochemistry and cell biology [2]. GFP and its mutant derivatives possess internal fluorophore that re-emits light which covers visible spectrum of colors [3]. Hence, GFP technology is very attractive and easily adaptable to biotechnology and many fields of basic science. GFP is used as a protein fusion reporter in various applications in diverse kind of cells for imaging and detection of very specific processes, such as fusion gene expression, tagged protein in situ location, host–parasite interactions and others [2]. The hybrid protein maintains its normal function along with fluorescent property acquired from GFP expressing gene. The incorporation of GFP can be done at either the N- or C-terminus of a protein or peptide domain.

Epigenetic effects of ribosomal and transcriptional frameshifting play important role in regulating gene expression processes as it has clearly emerged from the accumulated and still growing knowledge in this field [4]. Our research concerns comparative analysis of the transcriptional slippage propensity of the two most widely used RNA polymerases (RNAPs): *E. coli* and T7 bacteriophage, representing two different families of enzymes [5]. Long mononucleotide A/T stretches destabilize and realign RNA:DNA hybrid, contributing to RNAP “slippage” throughout the DNA template. Productive transcriptional slippage at such sites involves unwinding-rewinding of the RNA:DNA hybrid which is not sensed by RNAP active site and hereby does not lead to enzyme backtracking and mRNA correction. Forward and backward mRNA slippage generates insertion/deletion errors in nascent transcripts, resulting in shift of reading frame [6]. It is known that the error rate of transcripts generated by *E. coli* RNAP in vivo is significantly high, roughly between the values of 10^−5^ and 10^−3^ per residue [7], and even higher for T7 RNAP [6]. Insertion/deletion (indels) type of transcriptional errors may be considered as ambiguous. From one side they are detrimental to efficiency of gene expression, but on the other hand can also be beneficial for cell physiology. Transcriptional slippage has significant potential to restore the wild-type phenotype of indel mutant genes [6, 8–13]. Here, we evaluated GFP protein reporter for potential use in a study of the transcriptional slippage phenomenon which occurs during expression of out of frame fusion genes with upstream fragments containing A/T homopolymeric sequences. The results demonstrate utility of GFP fusion gene to study transcriptional slippage effect in homopolymer sequence-dependent manner.

## Methods

### Bacterial strains and culture conditions

All cloning was performed using *Escherichia coli* (*E. coli*) DH10B strain (F^−^ λ^−^ *mcr*A Δ(*mrr*-*hsd*RMS-*mcr*BC) Φ80*lac*ZΔM15 Δ*lac*X74 *rec*A1 *end*A1 *ara*D139 Δ(*ara leu*) 7697 *gal*U *gal*K *rps*L *nup*G) grown at 37 °C in Luria broth (LB) [14] supplemented with 50 μg/ml kanamycin or 100 μg/ml ampicillin for maintenance of pET24a- or pBAD24-based plasmids, respectively [15, 16]. For fluorescence experiments *E. coli* ER2566 T7 phage RNAP IPTG inducible strain (F^−^ λ^−^ *fhu*A2 [lon] *omp*T *lacZ*::T7 gene 1 *gal sulA*11 Δ(*mcr*C-*mrr*)114::IS*10* R(*mcr*-73::miniTn*10*-Tet^S^)2 R(*zgb*-210::Tn*10*-Tet^S^) *end*A1 [dcm]) was used (New England Biolabs, Ipswich, USA).

### Genetic techniques

Standard protocols [14] and kits were used for purification of plasmid DNA (A&A Biotechnology, Gdynia, Poland), digestion of DNA with restriction endonucleases, ligation of DNA with T4 DNA ligase and PCR techniques with PfuPlus DNA polymerase (all from EUR_x_, Gdansk, Poland).

### Vector construction

pET24a derivative reporter vectors (Additional file 1: Table S1) were constructed using PCR to amplify a 900-bp fragment of pGreenTIR plasmid [17], a source of enhanced fluorescence *gfp* variant (F64L/S65T) [18], and different set of primers (Additional file 1: Table S2) provided three reading frames (− 1, 0 and + 1). The resulting PCR fragment containing the promoterless *gfp* gene was digested with *Bam*HI and *Eco*RI and cloned into the same sites of pET24a. Next, sets of two annealed oligonucleotides (Additional file 1: Table S2) carrying appropriate polyA/T sequences were inserted into *Nhe*I–*Bam*HI sites and ligated. All resulting fusion genes were confirmed by sequencing (Genomed, Warszawa, Poland), and then, as *Xba*I–*Hin*dIII fragments were subcloned into pBAD24 plasmid.

### Site-directed mutagenesis

Site-specific mutagenesis using PCR was carried by high fidelity PfuPlus DNA polymerase (Eur_x_) according to the manufacturer’s instructions (50 ng of plasmid template was added to a 50-μl PCR reaction). Appropriate plasmid templates and nucleotide deletion/insertion in reverse primers were PCR-amplified and parental plasmid was eliminated by digestion with *Dpn*I enzyme (10 u, Thermo Scientific, Waltham, USA) for 1.5-h at 37 °C. Products were resolved in agarose gels, appropriate bands were cut out and aliquots containing purified DNA were transformed into DH10B competent cells. All plasmid modifications were confirmed by Sanger DNA sequencing using the BigDye Terminator v3.1 (Applied Biosystems, Waltham, USA) (Genomed). Additional file 1: Tables S1 and S2 include a list of plasmids and primers used.

### Whole cell fluorescence

ER2566 cells were grown in LB medium, containing kanamycin or ampicillin, at 37 °C, until the culture reached an OD_600_ of 0.2–0.3. Then, to induce expression of tested fusion genes isopropyl β-d-1-thiogalactopyranoside (IPTG) or l-arabinose were added to 1 mM or 0.1%, respectively, and cells were further incubated for additional 1 h. Cells were gently harvested (400 μl sample), resuspended in 200 µl F buffer (M9 salts; 0.1 mM CaCl_2_; 1 mM MgSO_4_) and then quantified using a Varioskan^®^ Flash Spectral Scanning Multimode Reader (Thermo Scientific) at excitation and emission wavelengths of 485 and 510 nm, respectively. The raw fluorescence intensity of each culture was normalized to cell density (OD_600_) and the background fluorescence from *gfp*-less cells was subtracted from each reading.

### Western blotting for GFP protein

Culture extracts, after normalization to OD_600_, were analyzed by 10% SDS-PAGE, transferred into nitrocellulose membrane and GFP protein detection was performed using mouse monoclonal anti-GFP (B-2) antibodies (Santa Cruz Biotechnology, Dallas, USA) diluted 1:4000 in TBS-T buffer (50 mM Tris–HCl, 150 mM NaCl, 0.05% Tween 20, pH 7.6) with 5% skimmed milk for 1.5 h at room temperature. After three washes with TBS-T, the membrane was incubated for 1 h with chicken anti-mouse IgG-HRP (horseradish peroxidase, 1:5000, Santa Cruz Biotechnology). The membrane was washed three times and the specific protein was visualized by adding chemiluminescent substrate solution (Pierce ECL Plus Western Blotting Substrate, Thermo Scientific) and exposed to X-ray film. For M2.MboII detection membrane was probed with rabbit anti-M2.MboII antibodies [19] diluted 1:1250 in TBS-T buffer, followed by incubation with a goat anti-rabbit secondary antibody conjugated with alkaline phosphatase (1:30,000, Sigma-Aldrich, Saint Louis, USA). A specific protein was visualized by adding BCIP/NBT substrate solution (Thermo Scientific).

### RNA extraction and cDNA synthesis

The cells were proceed as described in Whole cell fluorescence except after 1 h incubation with appropriate inducer cellular RNA was extracted with Total RNA Mini Plus Concentrator Kit (A&A Biotechnology) according to the manufacturer’s instructions. For the mRNA stability experiment culture samples were taken starting 30 s prior to addition of rifampicin (250 μg/ml, BioShop, Burlington, Canada). Culture sample volumes were corrected for OD to maintain similar cell numbers per sample. Samples were immediately mixed with 0.5 ml of stayRNA protection buffer (A&A Biotechnology). cDNA were obtained after RNase-free DNase I (Thermo Scientific) treatment by using RevertAid First Strand cDNA Synthesis Kit (Thermo Scietific).

### Northern blot detection

Profiles of the *gfp* fusion genes specific transcripts were analyzed by northern blotting. Equal amounts (5 μg) of total RNA were loaded on formaldehyde denaturing 1.3% agarose gel and then transferred onto Zeta-Probe^**®**^ blotting membrane (BioRad, Hercules, USA) by capillary forces. PCR-produced 774 bp dsDNA fragment specific to the whole *gfp* gene sequence (obtained with primers bamGFP6 and Gfpdown, Additional file 1: Table S2, and pGreenTIR as a template) after biotin labelling (Biotin-High Prime, Roche Diagnostics, Basel, Switzerland) was used as a probe. Chemiluminescent detection was carried using streptavidin-HRP Pierce ECL substrate (Thermo Scientific) and exposure to X-ray film.

### Quantitative RT-PCR

The specific primers, designed to ensure similar *T*_m_ and PCR product size, are given in Additional file 1: Table S2. 16S *rrn* was chosen as internal stable reference housekeeping gene, while in case of *gfp* fusion variants 5′-end of transcripts was analyzed. Real-time PCR with LightCycler 2.0 (Roche Diagnostics) was performed in triplicate in three independent experiments using SG qPCR mix with SYBR Green (Eur_x_). The PCR employed the following cycling parameters: 95 °C for 5 min, followed by 35 cycles of 94 °C for 20 s, 60 °C for 20 s, 72 °C for 10 s each; and finally the melting curve (60–97 °C) program for quality control, and cooling to 40 °C. The levels of remaining mRNA for each variant was normalized to the level of the reference housekeeping gene 16S *rrn*, and then determined by fitting the percentage of mRNA remaining vs. time to an exponential decay function. The relative fold-change mRNAs ratios were obtained by normalizing each time point data in reference to the earliest measurements. In all studies the half-lives of transcripts were determined by fitting the percentage of mRNA remaining vs. time to an exponential decay function. The relative fold-change mRNAs ratios were obtained by normalizing each time point data in reference to the earliest measurements [20].

### Analysis of the N-terminal amino acid sequence of the A_5_GFP-1 hybrid

The N-terminal protein sequence analysis was performed at BioCentrum Ltd. (Krakow, Poland). Sequentially detached phenylthiohydantoin derivatives of amino acids were identified using the Procise 491 (Applied Biosystems) automatic sequence analysis system, according to the standard protocol of the manufacturer.

## Results and discussion

### Contribution of a short and long N-terminal tags to the GFP fluorescence ability

**Fig. 1:**
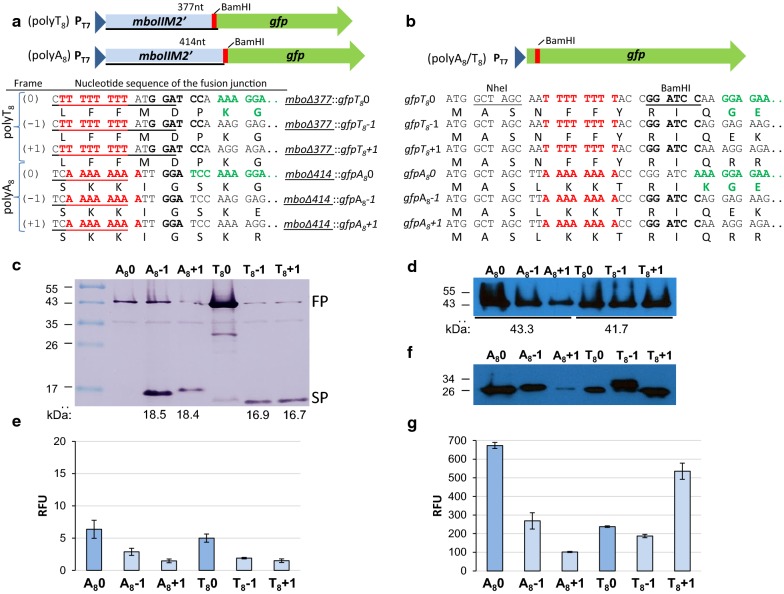
Effects of length of N-terminal tags on the expression and fluorescence of GFP hybrids. **a**, **b** Schematic representation and sequence details of a long (**a**) and short (**b**) tags with *gfp* in three reading frames (− 1, 0 and + 1). **c**, **d** Western blotting of long-tagged hybrids M2.MboIIΔ377::GFPT_8_ and M2.MboIIΔ414::GFPA_8_ detected with anti-M2.MboII (**c**) or anti-GFP antibodies (**d**), respectively. **f** Western blotting of short-tagged hybrids GFPA_8_ and GFPT_8_ with anti-GFP antibodies. *FP* full length products, *SP* short length products. **e** Relative fluorescence of the long-tagged hybrids; **g** relative fluorescence of the short-tagged hybrids. Standard deviations from three independent experiments are indicated

To determine how length and kind of nucleotides in the homopolymeric sequence affect the slippage events several fusions containing polyA and polyT sequences with downstream *gfp* gene [17] were constructed. Expression of fusion gene was under control of the T7-phage promoter [16]. First, fusions with short or a long (proximal half of *mboIIM2* gene [6]) fragments containing A_8_/T_8_ slippery sequences located upstream of the reporter *gfp* in three frames (− 1, 0 and + 1) were tested (Fig. 1a, b). Both type of gene fusion genes were properly expressed, however only in case of short N-terminal tags the fluorescence ability of the hybrid GFP was not disturbed (Fig. 1g), thus short fusion type was chosen to further studies.

### Identification of GFP reporter slippage product

**Fig. 2:**
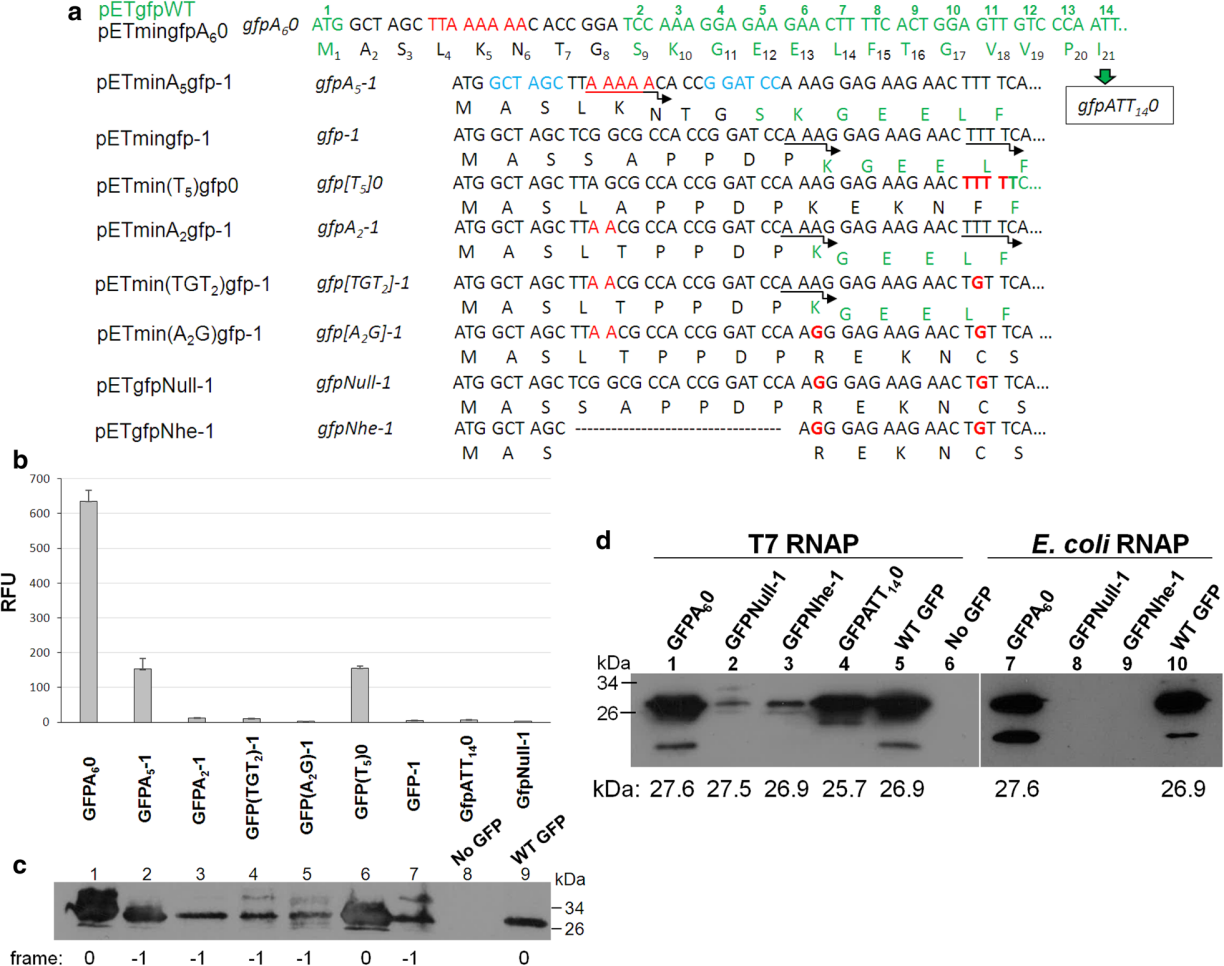
Evaluation of frameshifted (− 1) *gfp* fusion’s usefulness to serve as transcriptional slippage reporter. **a** Details of the nucleotide sequences of the proximal part of *gfp* fusions. The names of plasmid constructs’ and reporter gene variants, nucleotide sequences with possible sites of insertion slippage events (indicated by arrows), and primary and slippage-induced amino acid sequences are shown. Reading frame of genes (− 1 or 0) is reflected in their names as suffix 0 or − 1, respectively. Actual (black, below) and native (green, above) amino acid numbering of the GFPA_6_0 hybrid is provided. **b** Relative fluorescence level of indicated GFP hybrids. All measurements were performed in three to five duplicate repetitions. Error bars represent standard deviations. **c** Western blotting of the total cell extracts of hybrids shown in **b** and GFP immunodetection with ECL chemiluminescence system. **d** Extraordinary slippage properties of T7 RNAP in contrast to *E. coli* RNAP. ER2566 cells with appropriate pET24a-(lanes 1–5) or pBAD24-derived plasmids (lanes 7–10) carrying − 1 frameshifted *gfp* genes were induced with 1 mM IPTG or 0.1% l-arabinose at 37 °C, respectively. Aliquot of cell extracts were western blotted and immunodetected with anti-GFP primary antibodies. Below the molecular weight of the GFP protein products in kDa are provided

Our analysis was began with high slippage-prone sequence TTAAAAACACC [6]. pETminA_5_gfp-1 vector was constructed to contain PCR-created *gfp* gene in − 1 reading frame and an 18-bp duplex DNA carrying the homopolymer sequence inserted between *Nhe*I–*Bam*HI sites of pET24a vector (Fig. 2a), so as to apply the principle of avoiding the NGG codons [21]. Slippage efficiency of out-of-frame hybrid was indicated by the whole-cell fluorescence level referred to in-frame control. This in-frame *gfpA*_*6*_*0* fusion was designed to have the same nucleotide/amino acids pattern as the most likely product of erroneous single nucleotide insertion in *gfpA*_*5*_-*1*. Apart from 8 amino acid N-terminal tag this GFPA_5_-1 variant preserved all native GFP amino acids (S_2_K_3_G_4_E_5_E_6_L_7_…) and was capable of rescuing fluorescence up to 27% of the positive control level (Fig. 2b). We established that under our conditions observed fluorescence intensity was proportional to GFP hybrid yield (Additional file 1: Figure S1). To confirm whether GFPA_5_-1 hybrid protein was produced through site-specific slippage, the purified protein was subjected to N-terminal amino acid microsequencing by automated Edman degradation (BioCentrum, Poland). The sequence of the first 10 amino acid residues (A_2_S_3_L_4_K_5_N_6_T_7_G_8_**S**_9_**K**_10_**G**_11_) was found to be consistent with predicted after single adenine insertion into poly(A_5_) site (5A → 6A).

### Testing the proximal part of *gfp* gene for the possibility of an “surplus” slippage by T7 RNAP

To make sure that the detectable GFP fluorescence is owed only to the tested slippage sequence, the proximal part of *gfp* gene was examined for generating unexpected slippage or translational initiation from an alternative start codons. We checked possibility of production of truncated but functional GFP protein, by constructing − 1 frameshifted *gfp*-*1* fusion without any long slippery sequence (pETmingfp-1) and found it to manifest low fluorescence level, little above background (Fig. 2a, b). We reasoned that slight GFP protein production/activity was possible through slippage occurred at alternative sites located in the proximal part of *gfp*: AAA (27–29 nt) or TTTT (40–43 nt) (Fig. 2b, c). Such amazing ability of T7 RNAP to slip on the template containing at least three nucleotide repeats was already reported in in vitro experiments [22]. We confirmed possibility of bypassing single nucleotide deletion through slippage at T_4_ site by construction a pETmin(T_5_)gfp0 vector with in-frame *gfp* fusion gene “hopping” into the appropriate 0 frame by addition of a single T to the normally present four (15th amino acid). The resulting mutant variant GFP[T_5_]0, despite lacking 7 native N-end amino acids (M_1_A_2_S_3_L_4_A_5_P_6_P_7_D_8_P_9_K_10_E_11_K_12_N_13_F_14_**F**_15**(8)**_**T**_16**(9)**_**G**_17**(10**)_) exhibited 22% fluorescence compared to GFPA_6_0 positive control. Moreover, the importance of T_4_ run in restoration of the proper frame was confirmed by interruption of its continuity (*gfp[TGT*_*2*_*]*-*1*). Such a construct (pETmin(TGT_2_)gfp-1) showed decreased fluorescence by about 27% compared to pETminA_2_gfp-1 with full T_4_ run, yet its level was 4 times higher than background fluorescence (Fig. 2b, c). This suggested likely contribution of AAA sequence in slippage induction, what was further verified with a pETmin(A_2_G)gfp-1construct in which triple A sequence was modified (*gfp[A*_*2*_*G]*-*1*). Indeed, we observed subsequent decrease in GFP activity/production, but it reached little over the background.

To exclude the possibility, that fluorescent active GFP protein might be produced from a rare alternative start codon ATT [23], pETgfpATT_14_0 was constructed. In this variant *gfp* gene began from the 14th codon, which in the wild type is ATT, here substituted with ATG. Although we observed production of hybrid protein (Fig. 2d), yet no fluorescence was detected (Fig. 2b). This result is in agreement with data obtained by Raghunathan [24]. Interestingly, we detected remnant production of GFP even in case of − 1 frameshifted *gfpNull*-*1* and *gfpNhe*-*1* genes which lacked any triple A/T nucleotide in the 5′-terminal part of fusion gene (Fig. 2d). All of this indicates extremely high inclination of T7 RNAP to erroneous transcription, quite opposite to *E. coli* host RNAP (Fig. 2d).

### Relation between the intensity of hybrid GFP fluorescence and the number of retained natural amino acids

**Fig. 3:**
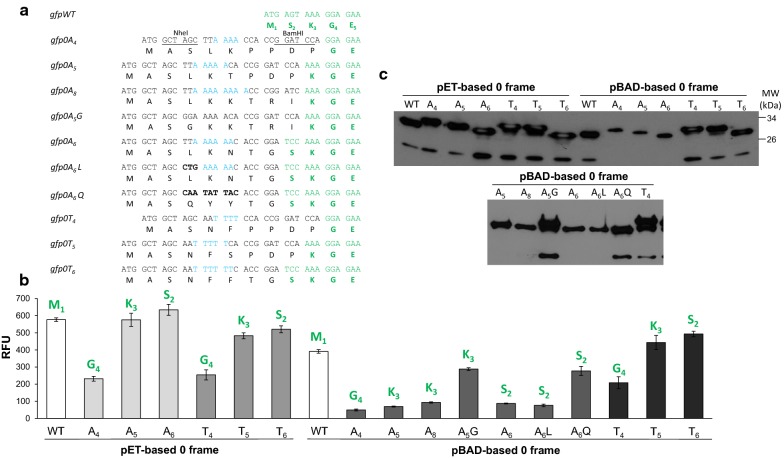
The relationship between the number of preserved natural N-terminal amino-acids and fluorescence intensity of the GFP hybrids. **a** Details of in-frame fusion genes sequence. **b** Relative whole-cell fluorescence of GFP hybrids generated by T7 and *E. coli* RNAP. The first preserved natural N-terminal amino-acid of GFP is marked. Standard error bars from at least three determinations are shown. **c** Levels of the poly(A) and poly(T)-based fusion gene expressions by immunodetection of GFP

**Fig. 4:**
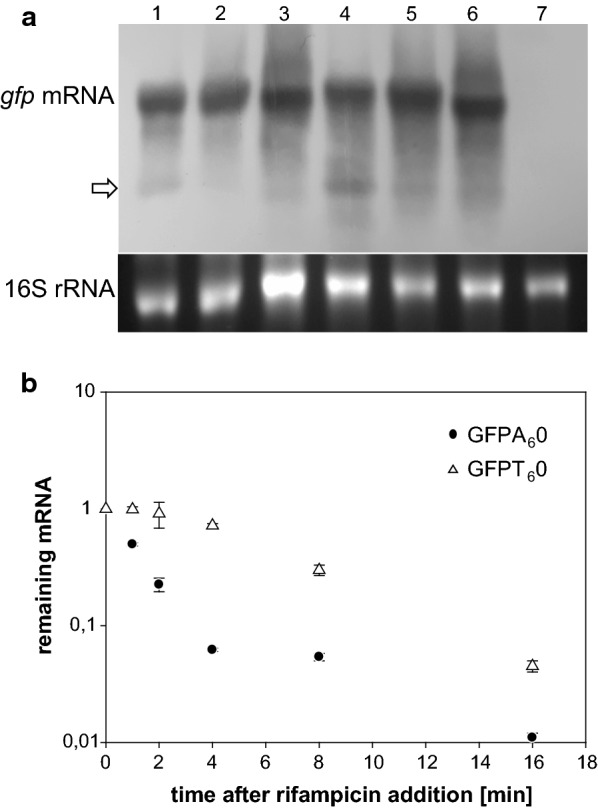
Analysis of polyA- and polyT-based *gfp* mRNA levels and stability. **a** Top panel: total mRNA was extracted from *E. coli* ER2566 bearing pBADmingfpA_6_0, pBADmingfpT_6_0 and pETmingfpA_6_0 plasmids after 10 min (lanes 1–3) and 30 min expression (lanes 4–6), respectively, generated by *E. coli* (lanes 1, 2, 4 and 5) or T7 phage RNAP (lanes 3 and 6). The membrane was probed with biotin labelled *gfp* DNA. Lane 7, no-*gfp* control bacteria. Arrow—prominent degradation product. Bottom panel: ethidium bromide-stained 16S rRNA are shown as loading control. **b** *gfp* transcripts stability. Rifampicin was added to an exponential culture of *E. coli* ER2655 growing in LB medium after 10 min of 0.1% L-arabinose induction. The mRNA levels were determined by RT-qPCR, using stable 16S rRNA as the internal standard. The circles represent *gfpA*_6_*0* and triangles represent *gfpT*_*6*_*0* transcripts. All mRNA levels were normalized to 1 at time = 0 (the points overlap). The data were fitted to an exponential decay. Standard error bars from three determinations are shown

Next, in order to examine fluorescence ability of GFP in-frame variants, a set of 0 frame fusion genes expressing GFP with various polyA/T tags was constructed (Fig. 3). Several studies determined tolerance of GFP protein to amino acid deletions at its N-terminus [25–29] and few to N-terminus insertions [30, 31]. However, to date, there is no universal rule indicating the impact of an additional amino acid at the N-terminus. As shown in Fig. 3, the relative levels of GFP hybrid fluorescence depended mainly on the number of retained natural amino acids, at least in the case of transcription driven by T7 phage RNAP. The lack of serine (S_2_) residue results in negligible negative effects (10%), while the lack of serine and lysine (S_2_K_3_) contributed to a significant reduction in fluorescence by 2.7-fold for polyA-based in-frame controls and twofold for polyTs. In the latter case, fluorescence level profile obtained with *E. coli* RNAP was quite similar, contrary to the polyA-based constructs, where for *E. coli* RNAP we observed a significant fivefold reduction in the fluorescence intensity (Fig. 3b). This is in agreement with low level of GFP production visualized by immunodetection (pBADgfpA_4_0-A_6_0, Fig. 3c). Since this effect occurred only for *E. coli* RNAP driven expression [32, 33], we ascribe it to the enzyme’s exceptional sensitivity to the mRNA regulatory region located downstream of the initiation codon (both constructs employ the same T7 RBS region), which impacts the translation speed resulting from interplay of multiple factors like codon bias, mRNA secondary structure, and co-translational protein folding coordination [31, 34–38]. Many studies have already shown that codon usage, codon pairs and their order in 5′-terminus are non-random and greatly influence protein production [21, 30, 39–45]. Therefore, we asked whether the rare leucine codon TTA_4_ could entail reduction of expression. It was replaced with five times more frequently used in *E. coli* CTG_4_ codon, but without any effect in the expression level. Whereas when it was exchanged for glycine codon GGA_4_ (*gfpA*_*5*_*G0*) threefold higher fluorescence was detected. Next, A-rich codons for L_4_K_5_N_6_ (TTA_4_ AAA_5_ AAC_6_) were substituted with other A/T-rich codon array of Q_4_Y_5_Y_6_ (CAA_4_ TAT_5_ TAC_6_) amino acids (*gfpA*_*6*_*Q0*). Those substituting amino acids are similar in their properties to N_4_F_5_F_6_ (AAT_4_ TTT_5_ TTC_6_) present in GFPT_6_0 hybrid, which exhibits high level fluorescence intensity. Indeed, in Q_4_Y_5_Y_6_ construct threefold higher fluorescence level was detected (Fig. 3b). Level of *gfpA*_*6*_*0*-construct mRNA was compared to *gfpT*_*6*_*0* and we found them equal both in northern blot analysis (Fig. 4a) as well as in quantitative RT-PCR (data not shown). We suggest that the codon context rule applies here in this specific pair of L_4_K_5_ codons [32, 33]. However, in case of *E. coli* RNAP generated *gfpA*_*6*_*0* samples prominent product of the full-length mRNA degradation was reproducibly observed (Fig. 4a, lanes 1 and 4 vs. lanes 2 and 5). To test whether differences in protein production level arise from differential mRNA stability, we measured the rate of decay patterns of each mRNA species following culture treatment with the transcription initiation inhibitor rifampicin (Fig. 4b). Indeed, polyA mRNA exhibited significantly shorter half-life than polyT (1 ± 0.001 min vs. 4.4 ± 0.13 min, respectively). Zucker MFold prediction program (mfold.rit.albany.edu) did not reveal any differences in local secondary structures between these two fusion species, thus, we suggest that uncoupling caused by codon specific context obstacles around the initiation region may contribute to more rapid mRNA decay, according to observations described elsewhere [37, 46]. Taking into consideration the complexity of several factors that impact efficiency of translation within a downstream of initiation codon sequence window and their interdependence ambiguity in creating stability of mRNA [47–51] we cannot exclude additional features that influence this rate-limiting initiation step of translation. Moreover, we reproducibly observed differences in migration pattern of the particular GFP hybrid produced by both T7 or bacterial expression systems, when using SDS polyacrylamide gels. Many of those did not correlate with their expected molecular weights, which are almost equal (27.6 kDa) to the wild-type GFP (26.9 kDa) (Fig. 2c, d). Presumably, this reflects differences in protein stability dependent on the amino acid composition of N-terminal tails.

### GFP reference normalization requirements

**Fig. 5:**
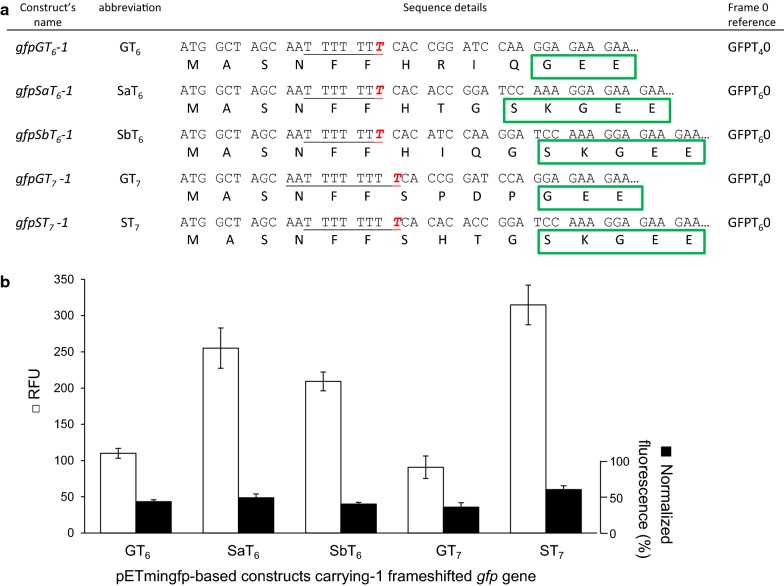
Slippage efficiency of the poly(T_6_/T_7_)*gfp*-*1* expressed genes presented as RFU and after normalization to fluorescence of corresponding GFP reference. **a** Sequence details of compared hybrids constructs with poly(T_6_) and poly(T_7_) (underlined) after transcriptional slippage by a single T insertion (italic red). **b** Fluorescence of hybrids shown in arbitrary units (white bars) and relative fluorescence values after normalization to fluorescence of a proper in-frame positive control values (black bars), respectively. Errors bars represent standard deviations from three independent experiments

The obtained results strongly indicate requirement of the proper positive reference with corresponding composition of natural amino acids to normalize fluorescence intensity of rescued frameshifted *gfp* mutants. In Fig. 5 fluorescence levels of variants of *gfpT*_*6*_-*1* and *gfpT*_*7*_-*1* series with various amino acid composition were presented. As shown (Fig. 5b) efficiencies of slippage are comparable within groups only after normalization to fluorescence of corresponding in-frame GFP0 controls (black columns vs. white columns).

## Conclusions

In this work, we present experimental evidence for utility of GFP reporter for accurate estimation of A/T homopolymeric sequence contribution in transcriptional slippage induction. We showed that the intensity of GFP fluorescence mainly depended on the number of retained natural amino acids, thus fluorescence raw data need to be referred to appropriate positive control. This GFP reporter-based tool can be easily applied to study of any slippage sequence.

## Additional file

**Additional file 1: Figure S1.** GFP fluorescence is proportional to a bacterial culture yield. The relative level of Gfp fluorescence in ER2566 cultures expressing 0 frame *gfp* (pETgfpA_6_0) (circles) and − 1 frameshifted *gfpA*_*5*_-*1* (pETminA_2_gfp-1) (diamonds) (1 mM IPTG by 1 h at 37 °C). The black line represents the regression line between normalized measurements of GFP fluorescence and bacterial pellet abundance, serially diluted (r^2^ = 0.998 and 0.992, as determined by regression analysis). **Table S1.** Plasmids used in this study. **Table S2.** List of oligonucleotides used in this study to amplify and duplex DNA annealing.
