# Supplementary material for: Evaluation of GFP reporter utility for analysis of transcriptional slippage during gene expression
Source: Microb Cell Fact. 2018 Sep 21;17:150. doi: 10.1186/s12934-018-0999-3 (PMC6149199; doi:10.1186/s12934-018-0999-3)
Supplement: Supplementary file 1 — Additional file 1: Figure S1. GFP fluorescence is proportional to a bacterial culture yield. The relative level of Gfp fluorescence in ER2566 cultures expressing 0 frame gfp (pETgfpA60) (circles) and − 1 frameshifted gfpA5-1 (pETminA2gfp-1) (diamonds) (1 mM IPTG by 1 h at 37 °C). The black line represents the regression line between normalized measurements of GFP fluorescence and bacterial pellet abundance, serially diluted (r2 = 0.998 and 0.992, as determined by regression analysis). Table S1. Plasmids used in this study. Table S2. List of oligonucleotides used in this study to amplify and duplex DNA annealing. [file 12934_2018_999_MOESM1_ESM.docx]

**Additional Figures and Tables**

**Evaluation of GFP reporter utility for analysis of transcriptional slippage during gene expression**

Wons E., Koscielniak D., Szadkowska M., and Sektas M.^*^

Department of Microbiology University of Gdansk, ul. Wita Stwosza 59, 80-308 Gdansk

*To whom correspondence should be addressed: Tel: (+4858) 5236068; Fax: (+4858) 5236073; Email: marian.sektas@biol.ug.edu.pl

# Figure S1. GFP fluorescence is proportional to a bacterial culture yield. The relative level of Gfp fluorescence in ER2566 cultures expressing 0 frame *gfp* (pETgfpA_6_0) (circles) and -1 frameshifted *gfpA_5_-1* (pETminA_2_gfp-1) (diamonds) (1mM IPTG by 1 h at 37^O^C). The black line represents the regression line between normalized measurements of GFP fluorescence and bacterial pellet abundance, serially diluted (r^2^=0.998 and 0.992, as determined by regression analysis).

**Table S1. Plasmids used in this study.**

| **Plasmid name** | **Relevant feature(s)** | **Reference** |
| --- | --- | --- |
| **pBAD24** | arabinose inducible *araBAD* promoter, *araC*, pBR322 ori, Amp^R^ | Guzman et al., 1995 |
| **pBADgfpWT** | pBAD24 derivative with SalI-SalI fragment bearing *gfp* gene from pGreenTIR plasmid | This work |
| **pBADmingfpA_4_0** | pBAD24 derivative with XbaI-HindIII subcloned fragment of pETgfpA_4_0 carrying translational fusion *gfp* gene with upstream A-rich fragment 5’-T_2_A_4_ (Fig. 2A) | This work |
| **pBADmingfpA_5_0** | pBAD24 derivative with XbaI-HindIII subcloned fragment of pETgfpA_5_0 carrying translational fusion *gfp* gene with upstream A-rich fragment 5’- T_2_A_5_ (Fig. 2A) | This work |
| **pBADgfpA_6_0** | pBAD24 derivative with XbaI-HindIII subcloned fragment of pETgfpA_6_0 carrying translational fusion of *gfp* gene with upstream A-rich fragment 5’- T_2_A_6_ (Fig. 2A) | This work |
| **pBADmingfpA_6_L0** | pBADmingfpA_6_0 derivative with 4th leucine codon TTA changed to the CTG codon | This work |
| **pBADmingfpA_6_Q0** | pBADgfpA_6_0 derivative with changed 4-, 5- and 6th codon for L_4_K_5_N_6_ amino acids to Q_4_Y_5_Y_6_ | This work |
| **pBADmingfpA_8_0** | pBAD24 derivative with XbaI-HindIII subcloned fragment of pETgfpA_8_0 carrying translational fusion of *gfp* gene with upstream A-rich fragment 5’-T_4_A_8_ (Fig. 2A) | This work |
| **pBADmingfpT_4_0** | pBAD24 derivative with XbaI-HindIII subcloned fragment of pETgfpT_4_0 carrying translational fusion of *gfp* gene with upstream T-rich fragment 5’-A_2_T_4_ (Fig. 2A) | This work |
| **pBADmingfpT_5_0** | pBAD24 derivative with XbaI-HindIII subcloned fragment of pETgfpT_5_0 carrying translational fusion of *gfp* gene with upstream upstream T-rich fragment 5’- A_2_T_5_ (Fig. 2A) | This work |
| **pBADmingfpT_6_0** | pBAD24 derivative with XbaI-HindIII subcloned fragment of pETgfpT_6_0 carrying translational fusion of *gfp* gene with upstream T-rich fragment 5’- A_2_T_6_ (Fig. 2A) | This work |
| **pBADminA_2_gfp-1** | pBAD24 derivative with XbaI-HindIII subcloned fragment of pETminT_2_A_3_gfp-1 carrying of modified -1 frameshifted *gfp* gene with reduced A-rich sequence (10-13 nt) 5’-TTAACGCCACC (Fig. 3A) | This work |
| **pBADminT_2_Agfp-1** | pBAD24 derivative with XbaI-HindIII subcloned fragment of pETminT_2_A_3_gfp-1 carrying of modified -1 frameshifted *gfp* gene with reduced A-rich sequence (10-12 nt) 5’-TTAGCGCCACC (Fig. 3A) | This work |
| **pBR322** | pMB1 ori, Amp^R^ Tet^R^ | Bolivar et al., 1977 |
| **pET24a** | Used for translational fusion with RBS and gene *10* of T7, IPTG inducible T7 promoter, pBR ori, Km^R^ | Novagene |
| **pETgfpNhe-1** | pET24a derivative with PCR created *gfp-1* gene inserted between NheI-HindIII (Fig. 1A) | This work |
| **pETgfpNull-1** | pETmingfp-1 and pETmin(A_2_G)gfp-1 derivative hybrid without polyA/T region with interrupted AA**G** (27-29 nt) and T**G**TT (40-43 nt) sequences (Fig. 1A) | This work |
| **pETgfpWT** | pET24a derivative with promoterless *gfp* gene from pGreenTIR inserted in SalI site (Fig. 2A) | This work |
| **pETmingfpA_4_0** | pET24a derivative with BamHI/EcoRI PCR created frame 0 *gfp* gene in translational fusion with upstream A-rich fragment 5’- T_2_A_4_ inserted between NheI-BamHI sites (Fig. 2A) | This work |
| **pETmboΔ377gfpT_8_0** | pET24 derivative with 377 nt proximal part of *mboIIM2* gene with polyT_8_ in translational fusion with *gfp* gene in 0 frame 0 (*mboΔ377::gfpT_8_0*, Fig. S2a) | This work |
| **pETmboΔ377gfpT_8_-1** | pET24 derivative with 377 nt proximal part of *mboIIM2* gene with polyT_8_ in translational fusion with *gfp* gene in -1 frame (*mboΔ377::gfpT_8_-1*, Fig. S2a) | This work |
| **pETmboΔ377gfpT_8_+1** | pET24 derivative with 377 nt proximal part of *mboIIM2* gene with polyT_8_ in translational fusion with *gfp* gene in frame 0 (*mboΔ377****::****gfpT_8_+1*, Fig. S2a) | This work |
| **pETmboΔ414gfpA_8_0** | pET24 derivative with 414 nt proximal part of *mboIIM2* gene with polyA_8_ in translational fusion with *gfp* gene in frame 0 (*mboΔ414::gfpT_8_0*, Fig. S2a) | This work |
| **pETmboΔ414gfpA_8_-1** | pET24 derivative with 414 nt proximal part of *mboIIM2* gene with polyA_8_ in translational fusion with *gfp* gene in -1 frame (*mboΔ414::gfpT_8_-1*, Fig. S2a) | This work |
| **pETmboΔ414gfpA_8_+1** | pET24 derivative with 414 nt proximal part of *mboIIM2* gene with polyA_8_ in translational fusion with *gfp* gene in +1 frame (*mboΔ414::gfpT_8_+1*, Fig. S2a) | This work |
| **pETmingfpA_5_0** | pET24a derivative with BamHI/EcoRI PCR created frame 0 *gfp* gene in translational fusion with upstream A-rich fragment 5’- T_2_A_5_ inserted between NheI-BamHI sites (Fig. 2A) | This work |
| **pETmingfpA_6_0** | pET24a derivative with BamHI/EcoRI PCR created frame 0 *gfp* gene in translational fusion with upstream A-rich fragment 5’-T_2_A_6_ inserted between NheI-BamHI sites (Fig. 2A) | This work |
| **pETmingfpA_8_0** | pET24a derivative with BamHI/EcoRI PCR created *gfp* in 0 frame in translational fusion with upstream A-rich fragment 5’- T_2_A_8_ inserted between NheI-BamHI sites (Fig. 2a, Fig. S2b) | This work |
| **pETmingfpA_8_-1** | pET24a derivative with BamHI/EcoRI PCR created *gfp* in frame -1 in translational fusion with upstream A-rich fragment 5’- T_2_A_8_ inserted between NheI-BamHI sites (Fig. S2b) | This work |
| **pETmingfpA_8_+1** | pET24a derivative with BamHI/EcoRI PCR created *gfp* in frame +1 in translational fusion with upstream A-rich fragment 5’- T_2_A_8_ inserted between NheI-BamHI sites (Fig. S2b) | This work |
| **pETmingfpT_4_0** | pET24a derivative with BamHI/EcoRI PCR created frame 0 *gfp* gene in translational fusion with upstream T-rich fragment 5’-A_2_T_4_ inserted between NheI-BamHI sites (Fig. 2A) | This work |
| **pETmingfpT_5_0** | pET24a derivative with BamHI/EcoRI PCR created frame 0 *gfp* gene in translational fusion with upstream T-rich fragment 5’-A_2_T_5_ inserted between NheI-BamHI sites (Fig. 2A) | This work |
| **pETmingfpT_6_0** | pET24a derivative with BamHI/EcoRI PCR created frame 0 *gfp* gene in translational fusion with upstream T-rich fragment 5’-A_2_T_6_ inserted between NheI-BamHI sites (Fig. 2A) | This work |
| **pETmingfpT_8_0** | pET24a derivative with BamHI/EcoRI PCR created *gfp* in 0 frame in translational fusion with upstream A-rich fragment 5’-A_2_T_8_ inserted between NheI-BamHI sites (Fig. S2b) | This work |
| **pETmingfpT_8_-1** | pET24a derivative with BamHI/EcoRI PCR created *gfp* in frame -1 in translational fusion with upstream A-rich fragment 5’-A_2_T_8_ inserted between NheI-BamHI sites (Fig. 1b) | This work |
| **pETmingfpT_8_+1** | pET24a derivative with BamHI/EcoRI PCR created *gfp* in frame +1 in translational fusion with upstream A-rich fragment 5’-A_2_T_8_ inserted between NheI-BamHI sites (Fig. 1) | This work |
| **pETgfpAAT_14_0** | pETgfpWT derivative with proximal deteted version of *gfp* gene beginning from alternative AUU start codon (changed to AUG), deprived of the first 13 N-terminal natural amino acids (Fig. 2A). | This work |
| **pETminT_6_Ggfp-1** | pET24a derivative with PCR created modified *gfp* gene -1 frameshifted without natural serine and lysine residues, in translational fusion with upstream homopolymer poly-T6 fragment , inserted between NheI and BamHII sites (Fig. 5A) | This work |
| **pETminT_6_Sagfp-1** | pET24a derivative with PCR created modified *gfp* gene -1 frameshifted retained all natural amino acids, in translational fusion with upstream homopolymer poly-T6 fragment, inserted between NheI and BamHII sites (Fig. 5A) | This work |
| **pETminT_6_Sbgfp-1** | pET24a derivative with PCR created modified *gfp* gene -1 frameshifted retained all natural amino acids, in translational fusion with upstream homopolymer poly-T6 fragment, inserted between NheI and BamHI sites (Fig. 5A) | This work |
| **pETminT_7_Ggfp-1** | pET24a derivative with PCR created modified *gfp* gene -1 frameshifted without natural serine and lysine residues, in translational fusion with upstream homopolymer poly-T7 fragment, inserted between NheI and BamHI sites (Fig. 5A) | This work |
| **pETminT_7_Sgfp-1** | pET24a derivative with PCR created modified *gfp* gene -1 frameshifted retained all natural amino acids, in translational fusion with upstream homopolymer poly-T7 fragment, inserted between NheI and BamHI sites (Fig. 3A) | This work |
| **pETmingfp-1** | pET24minAgfp-1 derivative with triple nucleotide changes (10-12 nt) 5’-GCGGCGCCACC (underlined) (Fig. 1A) | This work |
| **pETminA_5_gfp-1** | pET24a derivative with PCR created modified *gfp* gene -1 frameshifted, in translational fusion with upstream homopolymer poly-A5 fragment inserted between NheI and BamHII sites (Fig. 1A) | This work |
| **pETminAgfp-1** | pETminA_2_gfp-1 derivative with alteration of single A (13 nt) 5’-TTAGCGCCACC (underlined) (Fig. 1A) | This work |
| **pETmin(T_5_)gfp0** | pETminAgfp-1 derivative with insertion of additional T into 40-43 bp TTTT run of *gfp* gene creating frame 0 from 15th codon (*T_2_A[T_5_]0*) (Fig. 1A) | This work |
| **pETminA_2_gfp-1** | pET24a derivative with BamHI-HindIII PCR created modified *gfp* gene -1 frameshifted, in translational fusion with upstream fragment 5’- T_2_A_2_ inserted between NheI and BamHI sites (Fig. 1A) | This work |
| **pETmin(A_2_G)gfp-1** | pETmin(TGT_2_)gfp-1 derivative with change of single A on G (29 nt) in 5’-AAG (Fig. 1A) | This work |
| **pETmin(TGT_2_)gfp-1** | pET24minAgfp-1 derivative with change of single T (41 nt) in 5’-GTT (underlined) (Fig. 1A) | This work |
| **pGreenTIR** | IPTG inducible *lac* promoter with *gfp* from *Aequora victoria*, ori pUC, Amp^R^ | (Miller and Lindow, 1997) |
| **pUC18** | pUC ori, Amp^R^ | (Yanisch-Perron et al., 1985) |

**Table S2. List of oligonucleotides used in this study to amplify and duplex DNA annealing.**

| No | Name,  forward (up),  reverse (down) | Sequence (5’-3’) | Comment |
| --- | --- | --- | --- |
| 1 | 2A4T (up) | CTAGCAATTTTCCACCG | Oligos 1 and 2 were used to construct pETmingfpGT_4_0 (pBADmingfpT_4_0) plasmid |
| 2 | 2A4T (down) | GATCCGGTGGAAAATTG |  |
| 3 | 2A5T (up) | CTAGCAATTTTTCACCG | Oligos 3 and 4 were used to construct pETmingfpT_5_0 (pBADmingfpT_5_0) plasmid |
| 4 | 2A5T (down) | GATCCGGTGAAAAATTG |  |
| 5 | 2A6T (up) | CTAGCAATTTTTTCACCG | Oligos 5 and 6 were used to construct pETmingfpT_6_0 (pBADmingfpT_6_0) plasmid |
| 6 | 2A6T (down) | GATCCGGTGAAAAATTG |  |
| 7 | 2A6TS1 (up) | CTAGCAATTTTTTCACACCG | Oligos 7 and 8 were used to construct pETminT_6_S1gfp-1 plasmid |
| 8 | 2A6TS1 (down) | GATCCGGTGTGAAAAAATTG |  |
| 9 | 2A6TS2 (up) | CTAGCAATTTTTTCACATCCAAG | Oligos 9 and 10 were used to construct pETminT_6_S2gfp-1 plasmid |
| 10 | 2A6TS2 (down) | GATCCTTGGATGTGAAAAAATTG |  |
| 11 | 2AT7G (up) | CTAGCAATTTTTTTCACACACCG | Oligos 11 and 12 were used to construct pETminT_7_Ggfp-1 plasmid |
| 12 | 2AT7G (down) | GATCCGGTGTGTGAAAAAAATTG |  |
| 13 | 2T4A (up) | CTAGCTTAAAACCACCG | Oligos 13 and 14 were used to construct pETminA_4_gfp-1 and pETmingfpA_4_0 (pBADmingfpA_4_0) plasmids |
| 14 | 2T4A (down) | GATCCGGTGGTTTTAAG |  |
| 15 | 2T2A (up) | CTAGCTTAACGCCACCG | Oligos 15 and 16 were used to construct of pETminA_2_gfp-1, pETmin(TGT_2_)gfp-1 and pETmin(A_2_G)gfp-1plasmids |
| 16 | 2T2A (down) | GATCCGGTGGCGTTAAG |  |
| 17 | 2T1A (up) | CTAGCTTAGCGCCACCG | Oligos 17 and 18 were used to construct pETmin(T_5_)gfp0 plasmid |
| 18 | 2T1A (down) | GATCCGGTGGCGCTAAG |  |
| 19 | 2T5A (up) | CTAGCTTAAAAACACCG | Oligos 19 and 20 were used to construct pETmingfpA_5_0 (pBADmingfpA_5_0) |
| 20 | 2T5A (down) | GATCCGGTGTTTTTAAG |  |
| 21 | 2TA6 (up) | CTAGCTTAAAAAACACCG | Oligos 21 and 22 were used to construct pETmingfpA_6_0 (pBADmingfpA_6_0) |
| 22 | 2TA6 (down) | GATCCGTGTTTTTTAAG |  |
| 23 | 2TA6L (up) | CTAGC**CTG**AAAAACACCG | Oligos 23 and 24 were used to change 4th leucine codon CTG (in bold) to TTA, resulting in pETmingfpA_6_L0 (pBADmingfpA_6_L0) |
| 24 | 2TA6L (down) | GATCCGTGTTTTT**CAG**G |  |
| 25 | 2TA6Q (up) | CTAGCCAATATTACACCG | Oligos 25 and 26 were used to change three consecutive codons for leucine, lysine and asparagine amino acids (4th-6th) to glutamine, and two tyrosines (underlined), resulting in pETmingfpA_6_Q0 (pBADmingfpA_6_Q0) |
| 26 | 2TA6Q (down) | GATCCGTGTAATATTGG |  |
| 27 | ATT14gfp (f) | CCCCATGGTTGTTGAATTAGATGGTGATG | Primers 27 and 31 were used in PCR reaction to create proximal deleted version of *gfp* gene beginning from alternative AUU start codon (changed to AUG), deprived of the first 13 codons for natural amino acids (NcoI site). Resulted in pETgfpAAT_14_0 vector. |
| 28 | bamGFP4 (fwd) | GGGATCCAGGAGAAGAACTTTTCACTGGAG | Primers 28 and 31 were used in PCR to create *gfp* for pETmingfpA_4_0 and pETmingfpT_4_0 |
| 29 | bamGFP5 (fwd) | GGGATCCAAAAGGAGAAGAACTTTTCACTGGAG | Primers 29 and 31 were used in PCR to create *gfp* for pETmingfpA_5_0 and pETgfpT_5_0 |
| 30 | bamGFP6 (fwd) | GGGATCCAAAGGAGAAGAACTTTTCACTGGAG | Primers 30 and 31 were used in PCR to create *gfp* for pETmingfpA_6_0 and pETgminfpT_6_0 |
| 31 | Gfpdown (r) | CAGTGCCAAGCTTGCATGCCT | Reverse primer complementary to downstream of the *gfp* with HindIII site, carried on pGreenTIR plasmid (Miller and Lindow, 1997). |
| 32 | GfpTplus (f) | GAGAAGAACTTTT**T**CACTGGAGTTGTCC | Primers 32 and 33 were used in mutagenesis by PCR reaction to insert additional T nucleotide into 40-43 bp TTTT run of *T_2_Agfp*-1 gene on pETminT_2_Agfp-1 plasmid creating frame 0 from 15th codon (*[T_5_]gfp0* gene) |
| 33 | GfpTplus  (r) | GTG**A**AAAAGTTCTTCTCCTTTGGATCC |  |
| 34 | Nhegfp (f) | GGCTAGCAGGGAGAAGAACTGTTCACTG | Primers 34 and 31 were used in PCR reaction to create modified *gfp* gene in -1 frame in pET24a (NheI site). Resulted in pETgfpNhe-1 |
| 35 | rrnF | TCGGAATTACTGGGCGTAAAG | Primers 35 and 36 specific to 16S *rrn* genes were used in qPCR reaction generated DNA product of 160 bp |
| 36 | rrnR | CCTCCAGATCTCTACGCATTTC |  |
| 37 | RTgfpF | GGCCAACACTTGTCACTACTT | Primers 37 and 38 specific to 5’ proximal part of *gfp* were used in qPCR reaction generated DNA product of 156 bp |
| 38 | RTgfpR | GTGTCTTGTAGTTCCCGTCATC |  |
| 39 | TGf (f) | GGAGAAGAACTGTTCACTGGAGTTG | Primers 39 and 40 were used to change by PCR the second T to G in TT(G)TT sequence(40-43 bp) of *gfp* gene |
| 40 | TGr (r) | CAGTGAACAGTTCTTCTCCTTTGGATC |  |
| 41 | ZeroGFP (f) | CTAGCTCGGCGCCACCG | Oligos 41 and 42 were used to create gfp-1 fusion with fragment with no polyA/T (pETmingfp-1) |
| 42 | ZeroGFP (r) | GATCCGGTGGCGCCGAG |  |
| 43 | ZA8 | TTGGATCCAATTTTTTTTGATGAATTGGTTC | Oligo was used to construct of series (pETmboΔ414gfpA_8_) of fusion genes with terminal truncated *mboIIM2* containing polyA_8_ sequence. Restriction site BamHI is underlined |
| 44 | ZT8 | TTGGATCCATAAAAAAAAGCTTGGTGTTCTTCAG | Oligo was used to construct of series (pETmboΔ377gfpT_8_) of fusion genes with terminal truncated *mboIIM2* containing polyA_8_ sequence. Restriction site BamHI is underlined |

**Additional References**

Bolivar, F., Rodriguez, R.L., Greene, P.J., Betlach, M.C., Heyneker, H.L., Boyer, H.W., Crosa, J.H., Falkow, S. (1977) Construction and characterization of new cloning vehicles. II. A multipurpose cloning system. Gene **2**:95-113.

Guzman, L.M., Belin, D., Carlson, M.J., Beckwith, J. (1995) Tight regulation, modulation, and high-level expression by vectors containing the arabinose P_BAD_ promoter. J. Bacteriol. **177**:4121-4130.

[Miller, W.G., S.E. Lindow. (1997) An improved GFP cloning cassette designed for prokaryotic transcriptional fusions. Gene **191**:149-153.](http://cgsc.biology.yale.edu/Reference.php?ID=139320)

Yanisch-Perron C., Vieira C., Messing J. (1985) Improved M13 phage cloning vectors and host strains: nucleotide sequences of the M13mp18 and pUC19 vectors. Gene **33**:103-119.
